# Bayesian inference for low-rank Ising networks

**DOI:** 10.1038/srep09050

**Published:** 2015-03-12

**Authors:** Maarten Marsman, Gunter Maris, Timo Bechger, Cees Glas

**Affiliations:** 1 Psychometric Research Center, Cito, the Netherlands; 2 Department of Psychology, University of Amsterdam, the Netherlands; 3 Department of Research Methodology, Measurement and Data Analysis, University of Twente, the Netherlands

## Abstract

Estimating the structure of Ising networks is a notoriously difficult problem. We demonstrate that using a latent variable representation of the Ising network, we can employ a full-data-information approach to uncover the network structure. Thereby, only ignoring information encoded in the prior distribution (of the latent variables). The full-data-information approach avoids having to compute the partition function and is thus computationally feasible, even for networks with many nodes. We illustrate the full-data-information approach with the estimation of dense networks.

Modelling the joint distribution of binary variables is of importance in many fields of science, ranging from the study of phase transitions in statistical mechanics^1^ and the study of spatial statistics in biology^2^ to the study of comorbidity of mental disorder symptoms in psychiatry^3^. The Ising model^4^ is an appropriate model for such distributions, as it captures all main effects and pairwise interactions^5^.

Applications of the Ising model come in two distinct flavours, which are represented schematically in Figure 1 using the GNU-R package qgraph^6^. On the left hand side, variables interact only with their nearest neighbours. This is the situation for which physicists originally developed the Ising model. On the right hand side, every variable is (positively) correlated with nearly all other variables. This situation is typical for the social sciences^7^,^8^,^9^.

A key distinction between social science and physical science applications of the Ising model is in the rank of the connectivity matrix, which contains the strength of the pairwise interactions. Figure 2 presents eigenvalue spectra for each of the two networks in Figure 1. A near linear spectrum is found in the nearest neighbour network, whereas the eigenvalues in the dense network rapidly decrease in magnitude. As shown in the Methods section, the eigenvalues dictate the contribution of dimensions in the predictive distributions of variables in the network, and we have that the smaller eigenvalues in the dense network hold low predictive value in comparison with the larger eigenvalues. This observation suggests that the connectivity matrix of a dense network is well approximated by a low-rank matrix.

A vital property of low-rank approximations follows from a theorem published by Eckart and Young in the first volume of the journal Psychometrika^10^. The Eckart-Young Theorem shows that in a least-squares sense, the best approximation of rank *r* to a matrix consists of the eigenvalue decomposition in which all but the largest *r* eigenvalues are equated to zero. This theorem allows us to find a low-rank approximation to the full connectivity matrix, with the key property that the first eigenvalues and their corresponding eigenvectors can be recovered even if the remaining eigenvalues and eigenvectors are ignored. To demonstrate the power of this result, Figure 3 shows the rank four approximation to the two networks in Figure 1. As expected, the nearest neighbour network is not recovered in the rank four approximation, yet the dense network clearly is. This shows that this parsimonious low-rank approximation is useful to uncover the structure of the connectivity matrices of dense networks. In both cases, the Eckart-Young Theorem implies that the estimated eigenvectors in the low-rank approximation are the first few eigenvectors of the true connectivity matrix.

The connectivity matrix encoding the network structure is usually unknown and needs to be estimated from independent realisations of the network state. Estimating the network structure is difficult, however, because the likelihood is intractable and the number of unknown parameters is usually very large. As a solution, the pseudo-likelihood method^11^ has been developed. This method optimises each of the full-conditional (predictive) distributions of variables in the network, often in combination with regularisation constraints, either on the interactions in sparse networks and/or on the eigenvalues in dense networks. Here, we introduce an alternative that uses all available information from the data and not just the information in the full-conditional distributions. Although not the topic of this paper, this full-data-information method can be used in conjunction with regularisations of the types described above through a judicious choice of prior distributions.

The full-data-information approximation uses a latent variable representation of the Ising model that was developed by Kac^12^, further developed by Emch and Knops^13^ and independently discovered in many places in the statistical literature^2^,^14^,^15^,^16^,^17^,^18^. Specifically, every eigenvector for a connectivity matrix gives rise to a latent variable, such that all variables are independent conditionally on the full set of latent variables:
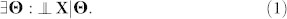
That is, there exist latent variables (Θ) that explain all the pairwise interactions in a statistical sense. The distribution of the variables conditionally on the latent variables is known as a multidimensional Item Response Theory (IRT) model in the field of psychometrics^19^, see the Methods section. The insight of Kac, Emch and Knops is schematically represented in Figure 4 using four latent variables. Ignoring some of the latent variables by equating the smallest eigenvalues to zero amounts to ignoring residual pairwise interactions but leaves the recovered eigenvalues and corresponding eigenvectors unaffected.

## Results

### Full-data-information estimation

The Ising model is mathematically elegant, yet notoriously difficult to compute. The main problem is the normalising constant *Z* in equation (5), called the partition function, which involves a sum over all 2*^n^* possible states of an *n* variable network. As the partition function depends on all of the model parameters, likelihood based statistical inference is impossible except for very small or severely constrained Ising models.

The computational problem becomes more tractable when we use the latent variable representation of the Ising model. The conditional distribution of the observed variables conditionally on the latent variables does not depend on the partition function and is available in an easily computed closed form. The partition function only figures in the distribution of the latent variables themselves. The posterior distribution of the Ising model parameters **W** (i.e., the connectivity matrix and main effects) and the latent variables ***θ*** given the data ***σ*** is proportional to:
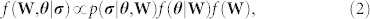
where *p*(***σ***|***θ***, **W**) and *f*(***θ***|**W**) are the multidimensional IRT model and latent variable distribution, as derived in the Methods section, and *f*(**W**) is a prior distribution for the model parameters. The whole computational complexity of this posterior distribution resides in the distribution of the latent variables, which depends on the model parameters and, in particular, on the partition function. Considering a Gibbs sampler^20^ for simulating from this posterior distribution, we find that the full conditional distribution *f*(***θ***|**W**, ***σ***) of the latent variables is highly tractable and does not involve the partition function, whereas the full conditional distribution *f*(**W**|***θ***, ***σ***) of the Ising model parameters is intractable because it involves the partition function.

When the latent variable distribution *f*(***θ***|**W**) in the Ising model is replaced by a prior distribution *g*(***θ***) that does not depend on the model parameters, we have a regular multidimensional IRT problem^19^:
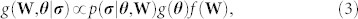
for which the full-conditionals *g*(***θ***|**W**, ***σ***) and *g*(**W**|***θ***, ***σ***) are easily sampled from. Multidimensional IRT models of this form are commonly applied in educational^21^ and psychological assessment^22^,^23^. Typically, the prior distribution *g*(***θ***) is a multivariate normal, and multidimensional IRT models with this choice of prior can be estimated using the MIRT package [www.utwente.nl/gw/omd/medewerkers/temp_test/mirt-manual.pdf].

It is clear that whenever *g*(***θ***) closely approximates *f*(***θ***|**W**), the much simpler problem in equation (3) can be used to infer about the problem in equation (2). Whether *g*(***θ***) closely approximates *f*(***θ***|**W**) is an empirical question. Figure 5 shows the (scaled) density *f*(***θ***|**W**) in the simplest nontrivial case, a fully connected network with all pairwise interactions equal to *a*, known as the Curie-Weiss model, for which *p*(***σ***|***θ***, **W**) is a Rasch model^24^. It is clear that the typically used normal approximation would work well for 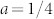; yet, when *a* increases, *f*(***θ***|**W**) first becomes skewed (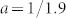) and ultimately becomes bimodal (*a* = 1) with modes drifting further apart as *a* increases.

In general, we cannot assume that the multivariate normal prior *g*(***θ***) closely approximates the Ising models latent variable distribution *f*(***θ***|**W**); thus, a different approach is needed. As we illustrate here and show in the Methods section, we can disregard the distribution of the latent variables *f*(***θ***|**W**) when we simulate from the conditional distributions of the Ising models parameters. In this way, all conditional distributions become tractable and, at the same time, all direct information on them provided by the observed variables is retained. The only information we ignore is that which is encoded in the prior distribution for the latent variables. That is, we combine the full-conditional of the latent variables from equation (2) with the full-conditional of the model parameters from equation (3). We call this approach full-data-information estimation.

We illustrate this using a simple Curie-Weiss model involving only pairwise interactions. This simplified model only involves one parameter, the unknown interaction strength *a*. In Figure 6, we see that the approximate posterior distribution nicely covers the true parameter value and becomes more concentrated around this value as the sample size increases.

### Data example

It is the combination of the Eckart-Young Theorem and the latent variable representation with full-data-information estimation that allows us to estimate low-rank Ising networks as we see them in the social sciences. To illustrate the approach, we consider a large educational measurement application. The Cito Eindtoets (www.cito.com) is a test consisting of 200 questions related to 12 theoretically distinct primary school subjects in arithmetic, language, and general study skills. The test is administered yearly to some 130, 000 children at the end of Dutch primary education. We present here the results from a rank three approximation to data from the 2012 Eindtoets.

Figure 7 displays both the rank three approximation and the individual rank one components as a heatmap. As argued above, even though the true connectivity matrix might be of a much higher rank than three, the three estimated components correspond to the three eigenvectors of the true connectivity matrix with the highest eigenvalues. The first component corresponds to a network in which all nodes are connected to one another, and (almost) all interactions are positive. The second and third component are such that particular sets of questions get higher positive interactions amongst themselves, whereas interactions between questions from different sets are negative. The second component is a contrast between the different language subjects (writing (W), spelling (S), and reading comprehension and vocabulary (RV)) and the subjects of mathematics (M) and study skills (SK). The third component is a contrast between the spelling subject and the other language subjects combined with the study skills subject. Note that, the positive interactions in one component can cancel the negative interactions in another component. For instance, mathematics and study skills have a positive interaction in the first and second component, whereas in the third component they have a negative interaction.

From the network replications (pupil responses) ***σ****_p_*, *p* = 1, …, *N*, we have that the matrix
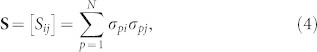
is sufficient for the connectivity matrix. We now study how the rank of the approximation impacts the prediction of **S**. To this aim, we perform a posterior predictive check and use the latent variable model 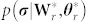 to generate new data, given draws 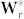 and 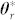 from the (partial-)posterior distributions (see the Methods section) of a rank *r* approximation; we then construct a new matrix 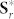 from the newly generated data. We show the residuals 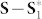 in Figure 8(a) as a heatmap, and similarly show the residuals 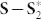 in Figure 8(b) and 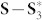 in Figure 8(c). In Figure 8(a), (b) and (c), we see that increasing the rank results in predicting more of the underlying structure of **S**. For instance, from Figure 7 we know that the second eigenvector captures the relation between mathematics and language items, and this structure in **S** is visible in the residuals 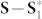 but not in the residuals 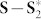.

Although higher rank approximations predict more of the structure in **S**, the rank one approximation already captures most of the variation in **S**. To see this, we plot the lower triangular elements of the matrix **S** against the lower triangular elements of 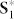 in Figure 8(d), against 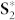 in Figure 8(e) and against 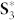 in Figure 8(f). The lower triangular elements of 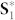 are highly correlated with the lower triangular elements from **S** (the correlation equals 0.995) with only minor improvements in the correlation for higher rank approximations. The correlation equals 0.997 in the rank two approximation and 0.998 in the rank three approximation.

## Discussion

We have shown how the Ising model could be estimated using full-data-information, in which we ignore prior structure on the parameters that resides in the latent variable model, and thereby effectively eliminate the need for computing the partition function from the statistical inference whilst retaining full-data-information. This approximate estimation technique opens the door for the estimation of other models, such as the Potts model^25^ or a mix of models for discrete and continuous random variables.

**Figure 2:**
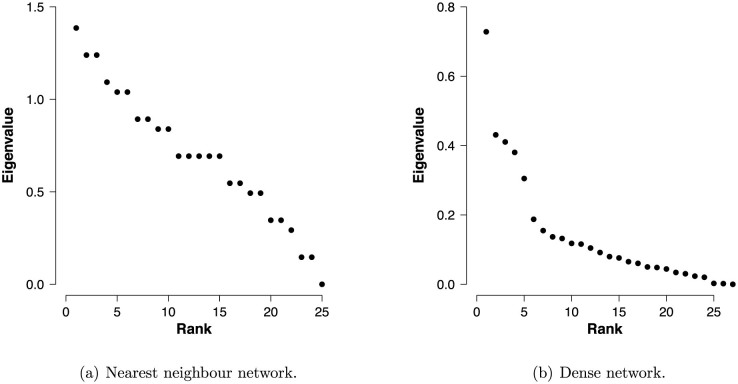
Key distinctions in eigenvalue spectra.

**Figure 5:**
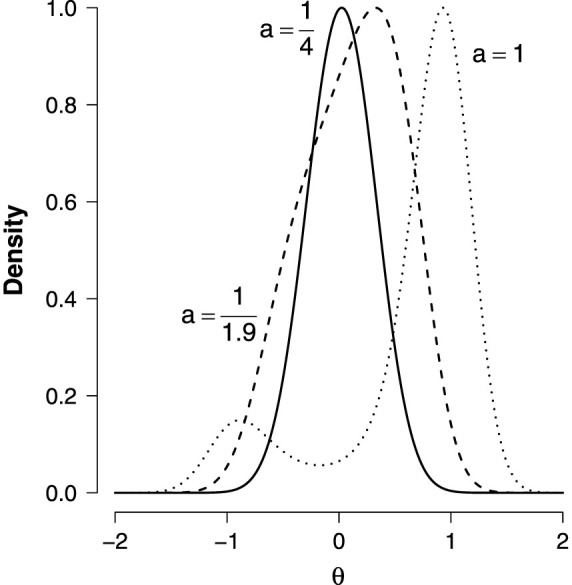
The scaled densities *f*(*θ*|W) in a Curie-Weiss model for 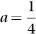 given as a solid line, 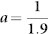 as a dashed line and 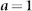 as a dotted line.

**Figure 6:**
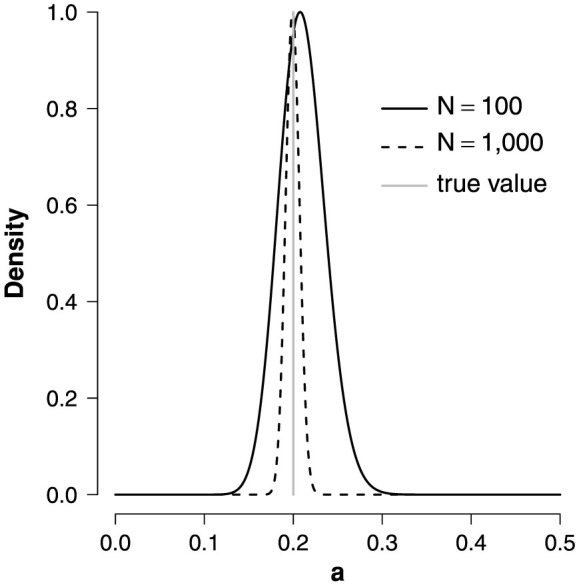
Partial conditional distribution of the interaction strength *a* in a Curie-Weiss model for *N* = 100 replications (solid line) and *N* = 1,000 replications (dashed line). The grey vertical line represents the true value.

**Figure 7:**
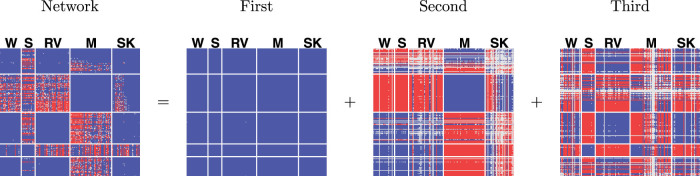
Heatmap of the connectivity matrix estimated in a rank three approximation to the 2012 Eindtoets data. The connectivity matrix is the sum of three rank-one matrices. Negative interactions are in red, positive interactions in blue, and small or absent interactions in grey.

**Figure 8:**
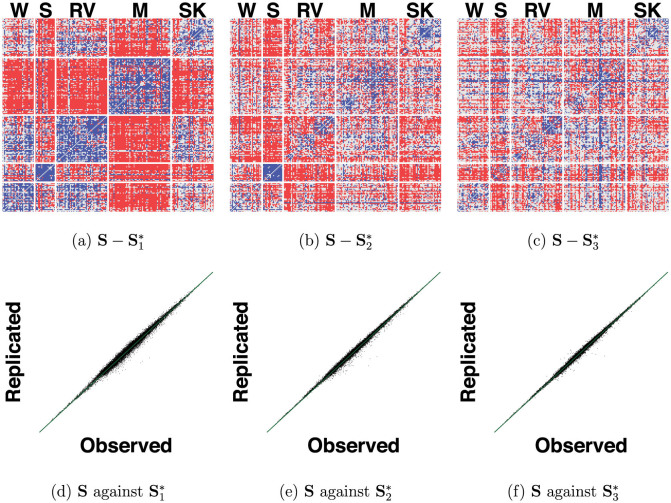
A heatmap of the matrix of residuals 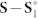 is shown in (a), that of 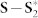 is shown in (b) and that of 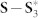 is shown in (c). Negative residuals are in red, positive residuals in blue, and small or absent residuals in grey. Also shown are the scatterplots of lower triangle elements of **S** against 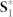 in (d), against 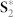 in (e) and against 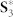 in (f). The green line is the first bisection.

As depicted in Figure 2(b), typical eigenvalue spectra found in the social sciences have a sharp drop in magnitude for the first few eigenvalues, after which the eigenvalues slowly decay. These plots resemble a mountain cliff with broken rock fragments at the base and are therefore called scree plots, where scree refers to the set of slowly decaying eigenvalues after the elbow. Scree plots are used to determine the relative importance of the eigenvalues, in which values after the elbow are often assumed ignorable due to, for instance, sampling error. However, the eigenvalues after the elbow in Figure 2(b) have a near linear spectrum, which resembles the spectrum found for the nearest neighbour network in Figure 2(a). This suggests that social science applications call for a mix of a dense network approximation, to use for the first few eigenvectors, and the nearest neighbour approximation of van Borkulo et al.^26^, to explain the residual structure.

The latent variable representation opens the way to new areas of research. For instance, the IRT model is closed under marginalisation, which shows that the IRT problem in equation (3) can be used as a starting point to approximate incomplete networks.

## Methods

### From Ising to Item Response Theory

The Ising model is characterised by the following distribution:
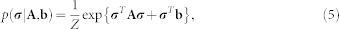
where the partition function *Z* serves to make the distribution sum to one and is a function of the main effects **b** and the connectivity matrix **A** containing the pairwise interactions. It is readily observed that all parameters are identifiable from the data, except for entries on the diagonal of the connectivity matrix.

Choosing diagonal values for the connectivity matrix such that all eigenvalues are non-negative, we obtain:
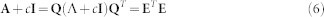
where **Λ** is a diagonal matrix with the eigenvalues of the original matrix **A**. In this expression, we conserve the off-diagonal entries in the connectivity matrix and, at the same time, ensure that the matrix **E***^T^***E** is positive (semi-)definite. This allows us to use the well known Gaussian identity to represent the Ising model equivalently in the following form:
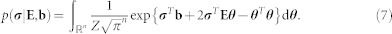
In this expression, the quadratic form is linearised, allowing for an explicit factorisation:
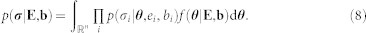
We can now recognise a multidimensional IRT model:
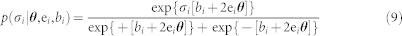
with a particular distribution for the latent variables:
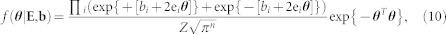
where **e***_i_* is the *i*-th row-vector of **E**. Note that, in this representation, the partition function figures as a normalising constant of the latent variable distribution.

### The full-conditional distribution *p*(*σ_i_* | *σ*_\*i*_)

The full-conditional distribution of a variable *σ_i_* given the other variables ***σ****_\i_* equals:
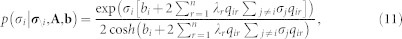
where 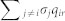 is recognised as the *r*-th principal component rest-score (i.e., the *r*-th principal component score minus *σ_i_q_ir_*). That is, *p*(*σ_i_* | ***σ*_\_**_*i*_, **A**, **b**) is a logistic regression model with intercept *b_i_*, principal component rest-scores as predictor variables and eigenvalues (times the *i*-th entry in the eigenvector) as regression coefficients.

### Full-data-information estimation

Upon choosing a proper prior distribution for the model parameters **E** and **b**, we obtain a posterior distribution for both the model parameters and the latent variables. It is not simple to simulate directly from this joint posterior distribution, thus we use the Gibbs sampler^20^. The full-conditional distribution of the latent variables corresponding to observation *p*, *p* = 1, …, *N*, is a multivariate normal distribution:
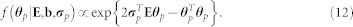
For the model parameters, we find more complicated full-conditional distributions that depend on the partition function. Because the partition function only figures as the normalising constant of the latent variable distribution, our proposal is to simulate the model parameters from the *partial* conditional distribution:
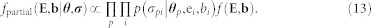
This partial conditional distribution ignores the information encoded in the marginal distribution of the latent variables but retains all the information about the model parameters that is contained in the data. This is why we refer to our approach as full-data-information estimation.

We alternately sample from the full-conditional distribution of the latent variables and the partial conditionals of the Ising model parameters. This does not amount to a proper Gibbs sampler in the sense that, after discarding the latent variables, the posterior distribution of the model parameters is not the invariant distribution. One way of looking at this scheme is that a second posterior distribution is set up for the latent variables and model parameters, one in which we have a proper prior distribution for the latent variables that does not depend on the model parameters. In this second posterior distribution, the partial full conditional distributions of the model parameters would be the correct full conditional distributions in a Gibbs sampler and it would be the multivariate normal full conditional distribution for the latent variables that would no longer be correct. When the Bayesian Central Limit Theorem would be the same for both posteriors, our scheme that mixes the full-conditional for the one posterior with another from the other posterior would still converge to the same Central Limit Theorem. In that sense, our full-data-information estimation admits the same large sample properties as does the full Bayesian estimation but without ever having to compute the partition function.

### Simulating from the partial conditionals

Simulating from the partial-conditional distribution of the parameters may seem to be a difficult problem in its own right, but this problem has been resolved in many different places^27^,^28^,^29^,^30^,^31^. We consider here a new method that is both computationally simple and highly efficient.

Our proposal is to use a Metropolis-Hastings algorithm^32^,^33^ for simulating from the partial-conditional distribution of the model parameters, which differs from earlier such approaches^27^,^28^ in the particular choice of the proposal distribution. We consider an independence Metropolis-Hastings algorithm^34^ in which the proposal distribution is easy to simulate from and the approximation of its target improves as the amount of data increases. This combination makes the algorithm ideally suited for large data sets.

Consider a set of random variables **Z**, such that 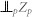 and *Z_p_* ~ *F_p_*. Define a matrix of binary indicator variables (coded as zero/one) with entries *x_pj_* = (*z_p_* < *z_j_*), such that for column *j* of the matrix we obtain:
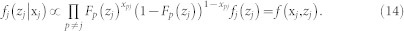
This distribution closely resembles the partial-conditionals. For any parameter *W*, the partial conditional is of the form:
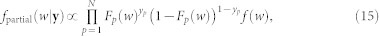
where *y_p_* = (*σ_p_* + 1)/2, *F_p_* is a (logistic) distribution function and *f*(*w*) the prior density of *W*. Thus, *f_j_*(*z_j_*|**x***_j_*) will be used as a proposal density.

To illustrate how the algorithm works, consider a simple case with *N* = 2 and a (target) partial conditional:
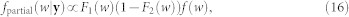
where 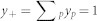. We now generate the vector **z** and choose *j* (in equation (14)) such that 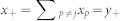 and find (for instance):
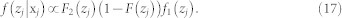
That is, *z_j_* is a draw from a posterior based on *N* observations and a total score *x*_+_, which differs from the target distribution w.r.t. the distribution of one of the observations and the prior density. In fact, the prior density and the distribution of the first observation have switched places.

That this method is feasible in practice is apparent from the Eindtoets data example. The Eindtoets data that we have used consisted of 200 variables (items) with 133,768 replications (pupils), and we considered a rank three approximation. A simple GNU-R implementation on a MacBook Pro with a 2.6 GHz Intel Core i5 processor (single core) took approximately 7 seconds to update the 200 unknown values in **b** and approximately 35 seconds to update the 600 unknown values in **E**. The average acceptance rate in 1,000 iterations of the Gibbs sampler was equal to 0.99 for the elements in **b** and 0.42 for the elements in **E**.

## Author Contributions

M.M. and G.M. wrote the main manuscript, T.B. and C.G. contributed to manuscript revisions, and M.M. prepared the figures. All authors reviewed the manuscript.

## Figures and Tables

**Figure 1:**
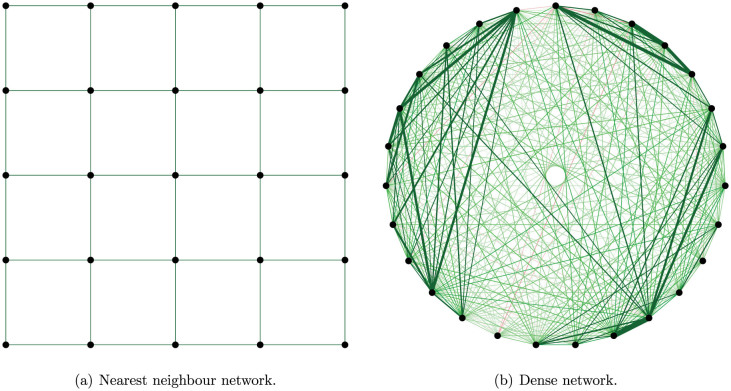
Two distinct flavours in applications of the Ising model.

**Figure 3:**
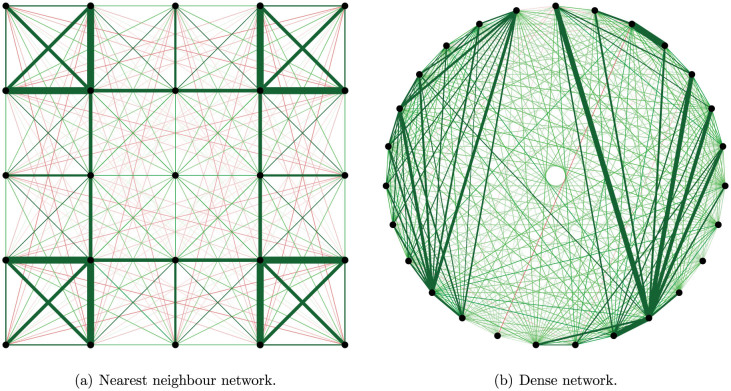
Rank four approximation to the networks in Figure 1.

**Figure 4:**
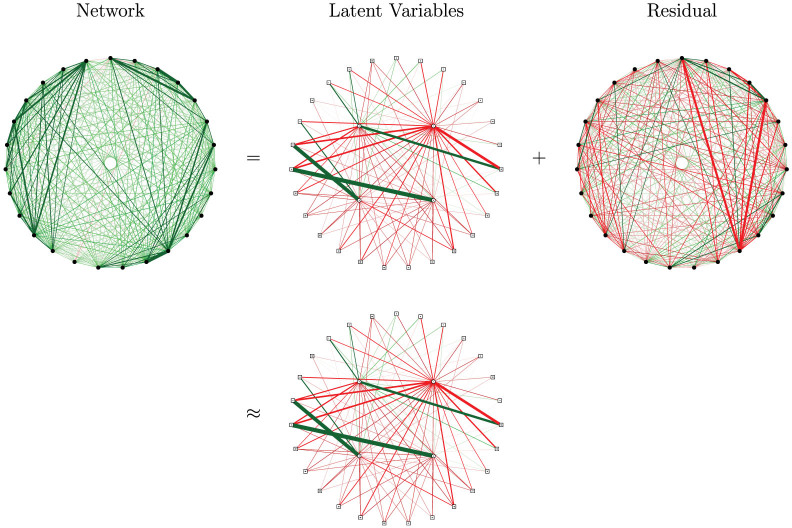
Rank four latent variable approximation to the dense network.
